# Book Review: Making Sense of Genes

**DOI:** 10.3389/fgene.2017.00124

**Published:** 2017-09-15

**Authors:** Tobias Uller

**Affiliations:** Department of Biology, Lund University Lund, Sweden

**Keywords:** science education, genetics, heritability, genetic determinism, genetic essentialism, history of genetics

The year is 2000. Bill Clinton, the President of the USA, stands on a podium. He is flanked by two scientists. They will soon announce the completion of a project that only a decade ago seemed impossible—the sequencing of the human genome. The human genome project was a huge success that transformed the life sciences. Yet, its greatest outcome was that it completely undermined the sales pitch for its own initiation. The banner behind the three men on stage announced that the genome was “The book of life” that could now be decoded to reveal the secrets of human development, health and disease—perhaps even the good and bad of our nature.

Today, in our excitement of what DNA sequencing allows us to do, it is easy to forget that none of those things actually have happened (and even easier to forget that many scientists thought those things *would* happen). But old habits die slowly. Scientific papers, undergraduate textbooks, popular science, magazines, blogs and tweets continue to refer to genes and genomes as programs, blueprints or recipes.

*Making Sense of Genes*, the new book by Kampourakis (2017), sets out to do what those metaphors are poorly equipped to do—to explain what genes are and what they are doing. Or, as Kampourakis makes a point of saying, what genes ARE NOT, and what they ARE NOT DOING.

Kampourakis, a Researcher of Science Education at the University of Geneva, is the right person for the task. Like his previous book, *Understanding Evolution, Making Sense of Genes* is a wonderfully engaging and pedagogical explanation of difficult concepts in biology. It fills a gap in the literature that—in hindsight—seems highly conspicuous. Whereas, books on genes and genomes for a lay audience glut themselves on ambiguous metaphors, undergraduate texts are technical and rarely spend more than a few sentences on conceptual issues. The philosophy of biology literature has many gems, but to the biology student these may seem distant and, well, too *philosophical*.

Kampourakis has an incredible feeling for how to strike the balance between biological material and conceptual analysis. Never losing sight of the science or molecular details, he moves in and out of philosophy through seams that are barely noticed. This will make the book a very rewarding read for many, but it is particularly suitable for undergraduate students in the life sciences. It would work eminently alongside traditional lecture-based teaching, as material for discussion groups, or as a means to advance students' understanding of genetics in courses that follow a more evidence-based learning method. If you are teaching life sciences or engaging in any form of public outreach, this book is a must-read.

The book begins with a discursive introduction, followed by three largely historical chapters that outline how the gene concept has changed over time. Introductions may receive only a passing glance by readers, but this one is crucial as it tells the reader about the road map and the destination for the historical chapters to come. As such, it would have benefitted from another page or two, especially since students in the natural sciences are not used to the idea that we need to look at the past to understand the present. Chapter four takes stock, and gives a clear and concise explanation of the current understanding of what genes are.

One may now expect Kampourakis to get into the gory details of what genes do. Not so. The three chapters that follow are devoted to the notion that there are “genes for” characters. This is an excellent example of how Kampourakis teaches and writes—weaving together the science of genetics, conceptual issues, and societal significance. By exposing the problems with thinking and talking about “genes for,” the relevance of the historical material comes to life and the reader is—perhaps uncomfortably!—forced to reflect on her own views. Is there perhaps some genetic determinism lurking underneath our interactionist assurances?

Only now does the author return to what genes do. First out is development. In an uncharacteristic reliance on metaphor, Kampourakis follows his debunking of the genome as a program with explaining that it should be seen as a plan. The origami example is illuminating, but reveals a tension. Is it not that, in biological development, the folding of the paper is part of the explanation for the appearance of the instructions for the next fold? Like the recipe metaphor, the genome-as-a-plan seems to assign causal and informational privileges that are at odds with the author's general stance. Maybe here was a missed opportunity to reflect on the use of metaphor in scientific explanation, a topic deferred to the concluding remarks. This quibble aside, I fully agree that development is the right context for genetics, rather than the other way around (as is commonly how the topics are taught today).

The second thing that genes do, Kampourakis tells us, is to account for a part of heritable variation in characters. This chapter includes an excellent explanation of heritability as well as an illuminating discussion of what it means for a gene, or anything else, to be a difference maker. Next comes epigenetics, a difficult topic that, after genetics, may be the main source of confusion and hyperbole in contemporary biology. Kampourakis steers admirably clear of the pitfalls and provides a clear and critical analysis. Throughout, the material is wonderfully up to date. The book ends with the prospects of genome sequencing in medicine. Would you want to know if you carry an allele that is associated with cancer? Kampourakis shows that answering this questions will be helped by an understanding not only of what genes do, but also of probabilities.

Throughout, Kampourakis reveals how science enters society via interpretation, and that scientists' interpretations may (often will) not be the same as that of the public. The pedagogical challenges are great, but this important book will help us to explain genetics to our friends, students, and fellow academics.

## Author contributions

The author confirms being the sole contributor of this work and approved it for publication.

### Conflict of interest statement

The author declares that the research was conducted in the absence of any commercial or financial relationships that could be construed as a potential conflict of interest.
